# Experimental Study on the Grinding of an Fe-Cr-Co Permanent Magnet Alloy under a Small Cutting Depth

**DOI:** 10.3390/mi13091403

**Published:** 2022-08-26

**Authors:** Ningchang Wang, Feng Jiang, Jianhui Zhu, Yuchun Xu, Chaoyu Shi, Heliang Yan, Chunqing Gu

**Affiliations:** 1 Zhengzhou Research Institute for Abrasives & Grinding Co., Ltd., Zhengzhou 450001, China; 2 State Key Laboratory of Super-Abrasives, Zhengzhou 450001, China; 3 Institute of Manufacturing Engineering, Huaqiao University, Xiamen 361021, China; 4 National & Local Joint Engineering Research Center for Intelligent Manufacturing Technology of Brittle Materials Products, Xiamen 361021, China

**Keywords:** Fe-Cr-Co permanent magnet alloy, grinding, small cutting depth, surface morphology, surface roughness

## Abstract

A small cutting depth is the key parameter to realize precision in the machining process. The stability of the machining process will directly affect the quality of machining. In this study, dry grinding experiments using an Fe-Cr-Co permanent magnet alloy with small cutting depths (5 μm) were carried out. The relationship between the number of peaks and valleys and the quality control of the grinding force, wheel speed and feed speed were analyzed. The relationship between the peak and valley values of the grinding force signals and the peak and valley values of the grinding surface obtained using a white light interferometer was revealed. The influence of the grinding parameters on the grinding forces was analyzed by processing the grinding force signals with a low-pass filter based on the rotational speed of the grinding wheel. The experimental results indicated that the difference in grinding force between the peak and valley could be reduced by increasing the grinding wheel speed, which was mainly due to a decrease in average grinding force when the maximum undeformed cutting thickness of the single abrasive decreased. The actual height difference between the grinding surface peak and valley could be realized by increasing the grinding wheel speed. The feed speed of the worktable had no effect on the grinding force signal and the peaks and valleys of the surface morphology. Lower surface roughness could be achieved by reducing the feed speed and increasing the grinding wheel speed.

## 1. Introduction

Fe-Cr-Co permanent magnet alloys not only have excellent machinability and good magnetic performance, but also possess good ductility compared to ferrite, Nd-Fe-B and other brittle permanent magnet materials [1,2,3,4,5]. Furthermore, their corrosion resistance and temperature stability are also excellent [6,7]. Therefore, Fe-Cr-Co permanent magnet alloys are widely used in engineering [8,9,10]. In recent years, with the development of science and technology, the machining precision of magnetic materials has become more and more important. Most investigations have mainly been focused on the effect of various factors on the magnetic properties and structure of Fe-Cr-Co permanent magnet alloys, including heat treatment processes [11,12,13], temperature and mechanical treatment [14,15,16,17,18] and changed alloy elements [19,20,21,22]. For example, Wu Xin et al. found that alloy treated with solid solution and thermal magnetic and multi-step aging exhibited better magnetic properties when saline water was used as the solution coolant [11], while Liu Yan et al. found that Fe-Cr-Co permanent magnets under the action of low-frequency vibration force, there would be a regular demagnetization increase phenomenon, but vibration to a certain time, the magnetic properties basically tend to constant.[15]. Furthermore, E. V. Belozerov et al. reported hard magnetic (Fe-22% Cr-15% Co) alloys with high strength and plasticity, and sufficiently high magnetic properties were obtained by the doping of alloys with tungsten and gallium [20,21]. However, few investigations on the effect of grinding on the performance of Fe-Cr-Co permanent magnetic alloys have been reported. Therefore, research on the grinding of Fe-Cr-Co permanent magnet alloys provides an important industrial contribution.

The influence of grinding parameters on grinding forces and surface quality when processing an Fe-Cr-Co permanent magnetic alloy under a small cutting depth was investigated in this study. The waved grinding force signals produced under a small cutting depth are unavoidable due to the runout of the grinding wheel. Therefore, under these conditions, it is particularly difficult to process the original force signal reasonably. Most scholars use a low-pass filter and then take the average of the original signals as the processing method, which cannot really reflect the relationship between the actual depth of the cut and the grinding forces, especially in the case of a small cutting depth. Therefore, the main focus of this paper was to study the method of processing forces and discuss the relationship between the actual cutting depth and grinding forces.

## 2. Experiments

### 2.1. Workpiece Materials

**Figure 1:**
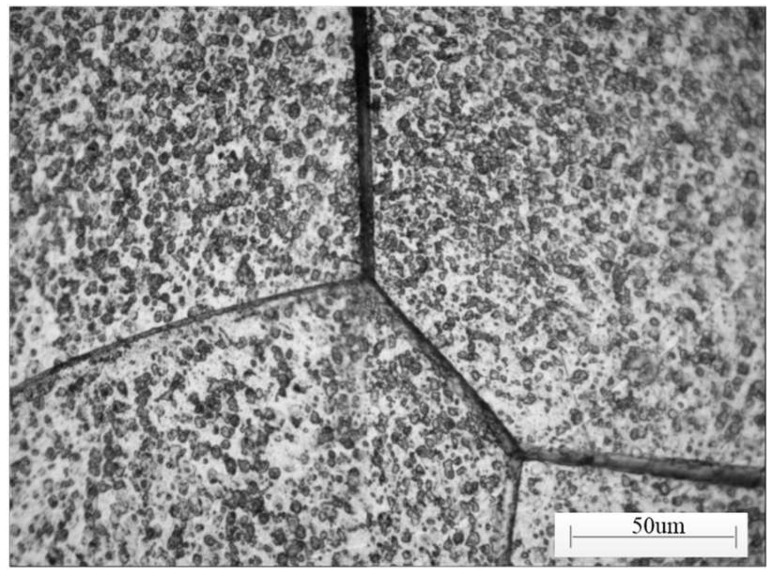
Optical microstructure of Fe-Cr-Co permanent magnet alloy.

The heat treatment state of the workpiece material included solution treatment, magnetic field heat treatment and three-stage tempering. An optical image of the microstructure of the Fe-Cr-Co permanent magnet alloy is shown in Figure 1. The chemical elements and mechanical properties of the Fe-Cr-Co permanent magnet alloy are listed in Table 1 and Table 2, respectively.

### 2.2. Grinding Experiments

**Figure 2:**
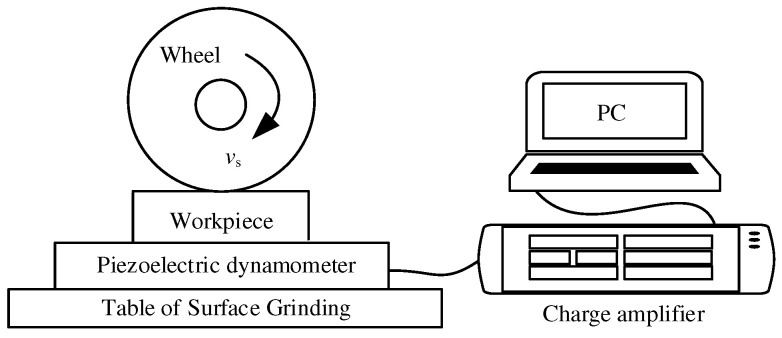
Illustration of experimental setup for grinding.

A high-speed surface grinder (HP408, BLOHM-Planomat, Hamburg, Germany) was used for the investigations. The motion system of the machine tool is equipped with a grating ruler, whose precision is 0.01 μm. Figure 2 illustrates the setup of the grinding experiment. A white corundum grinding wheels (PA46L, Saint Gobain, Shanghai, China) was chosen for the experiment. Single factor experiments (3 × 4) were used in this study. The workpieces were treated with several finishing processes before the experiment in order to remove surface irregularities, burrs and other defects in the workpiece. The grinding wheel was dressed using a diamond pen before each experiment. The purpose of dressing the grinding wheel before each machining was to avoid the influence of grinding wheel wear and surface integrity on the results. It should be emphasized that the dressing of the grinding wheel was carried out under the condition of low speed, and the load of wheel dressing was particularly small. Therefore, the contour or concentricity of the grinding wheel would not change during dressing. The grinding forces were measured with a piezoelectric dynamometer (9119AA2, Kistler, Winterthur, Switzerland) with an acquisition frequency of 5kHz. Each experiment was repeated six times and the average values of the experimental results were used as the research objects. The design of the grinding experiments is listed in Table 3.

**Figure 3:**
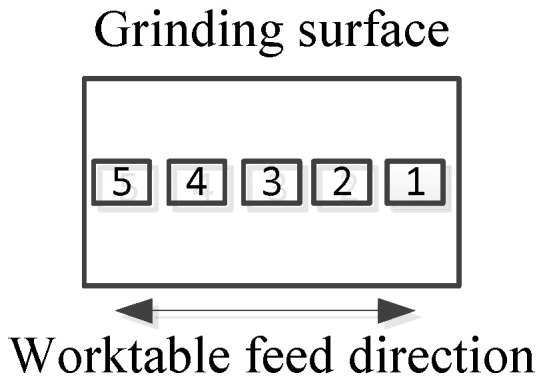
Schematic diagram of measuring position for ground surface roughness.

The surface morphology of the specimens after grinding was investigated using a white light interferometer. The ground surface roughness was measured by interval sampling five points along the worktable feed direction; each sampling point area was 1.41 mm × 1.06 mm, where 1.41 mm was the length in the worktable feed direction and 1.06 mm was the length in the wheel width direction. In this study, the roughness of the sample was characterized by the average of five sampling points; the measured positions are shown in Figure 3.

## 3. Results and Discussions

### 3.1. Grinding Force Signals

**Figure 4:**
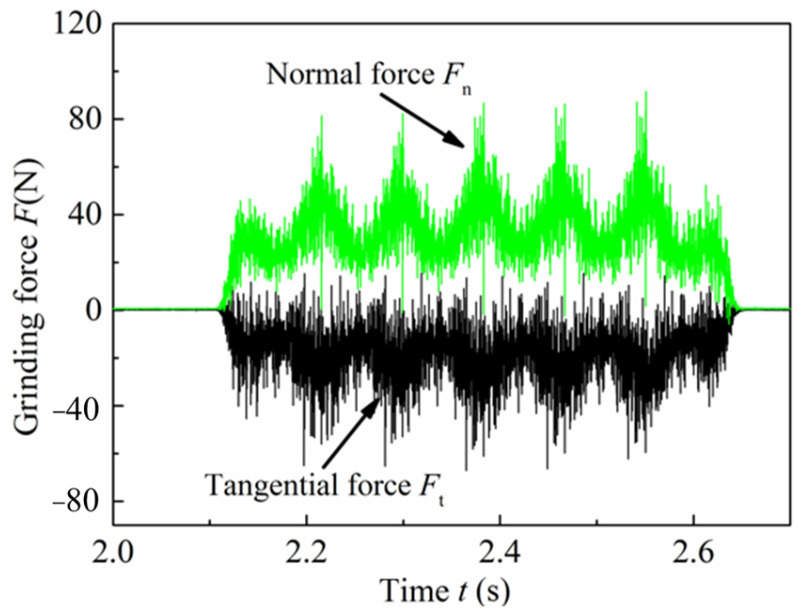
The typical grinding force signals without filtering.

**Figure 5:**
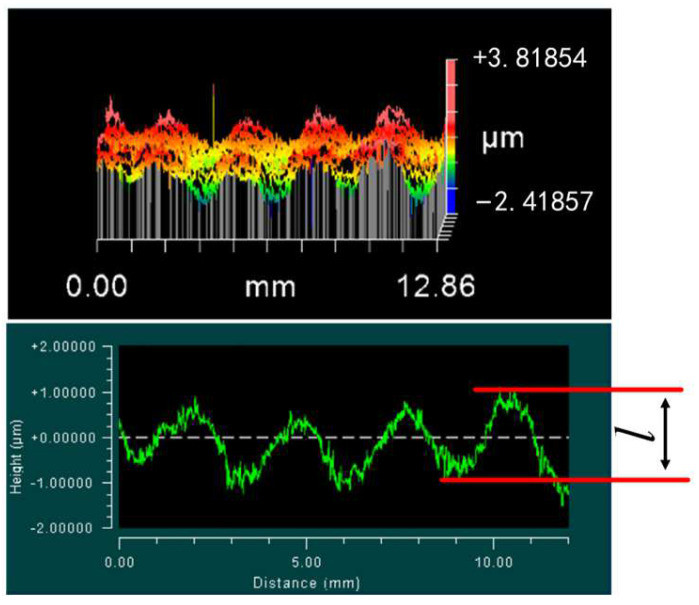
Typical image of surface morphology after grinding.

The typical grinding force signals without filtering are shown in Figure 4. The grinding signal consisted of three stages, including cut in, steady and cut out stages. The grinding force changed in sinusoidal wave form at the stable stage. The three stages of the grinding signals are presented in the different grinding process parameters. In order to analyze the formation mechanism of the above characteristics, the surface morphology after grinding was observed; a typical image is shown in Figure 5.

It can be seen from Figure 4 and Figure 5 that the grinding force curves were consistent with the cross-sectional contour of the ground surface morphology. This result was mainly caused by the radial runout of the grinding wheel. The peak and valley values of the grinding forces correspond to the valley and peak positions of the surface morphology, which are the maximum and minimum cutting depths in the grinding process, respectively. To further confirm that this result was caused by radial runout, the following method was used: calculate the number of all peaks in the normal force *Fn*, represented by the symbol *u*, and then analyze the relationship between *u* and *z*

*n* = *v_s_*/π/*D* (1)

*t* = 60 × *L*/*v_w_* (2)

*z* = *n* × *t* (3)

where *n* is the rotational speed of the grinding wheel (rev/s), *D* is the diameter of the grinding wheel (mm), *v_s_* is the wheel peripheral speed (m/s), *L* is the grinding length of the workpiece (mm), *v_w_* is the worktable feed (mm/min), *t* is the time of workpiece grinding (s) and z is the time of wheel runout, which is theoretically within the grinding length of workpiece. The relationship between u and *z* can be calculated using Equations (1)–(3). A comparison between *u* and *z* is shown in Table 4. The value of *z* was consistent with that of *u*, which further confirmed that the grinding signal was mainly caused by the radial runout of the grinding wheel.

### 3.2. Surface Morphology and Data Processing

**Figure 6:**
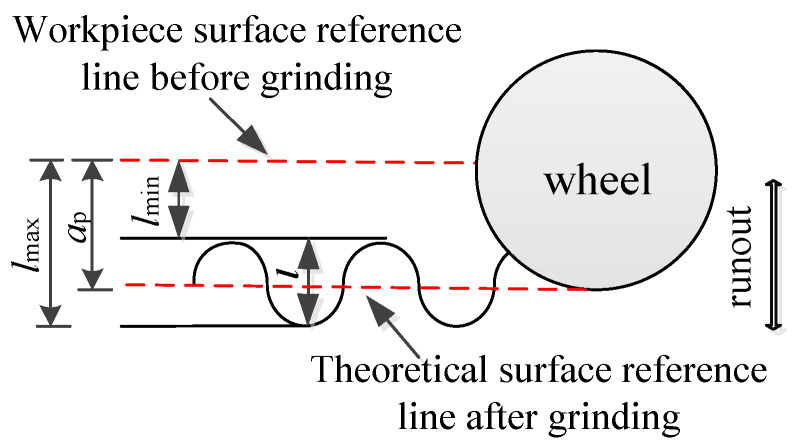
Sketch of surface morphology during the grinding process.

It can be seen from the above analysis that the grinding force signal inevitably fluctuated under the condition of a small cutting depth due to the runout of the grinding wheel. Therefore, grinding force data should be processed by combining the grinding surface topography and filtering frequency. In this study, the single-value grinding wheel speed *n* (rev/s) was selected as the filtering frequency, and the relationship between the actual cutting depth and grinding force was analyzed combined with the grinding surface topography. The surface topography in the grinding process is shown in Figure 6. In Figure 6, *l*_max_ and *l*_min_ represent the maximum and minimum values, respectively, of the actual grinding depth and *l* represents the difference between the peaks and valleys of the ground. The peak–valley value (PV), which could be obtained from the ground morphology, is equal to the *l* value in the profile, as shown in Figure 5. In the figure, *a_p_* is the cut depth, which was 5 μm in this study.

This relationship can be expressed as shown in Equation (4):

*l*_max_ + *l*_min_ = 2 × *l*_min_ + *l* = 2 × *a*_p_ (4)

The values of *l*_max_ and *l*_min_ are shown in Table 5.

### 3.3. Influence of Grinding Parameters on Grinding Forces

**Figure 7:**
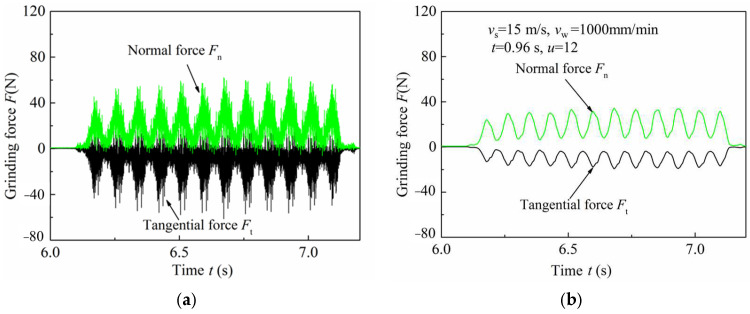
Curves of grinding force signal: (**a**) unfiltered; (**b**) filtered by 12 Hz.

**Figure 8:**
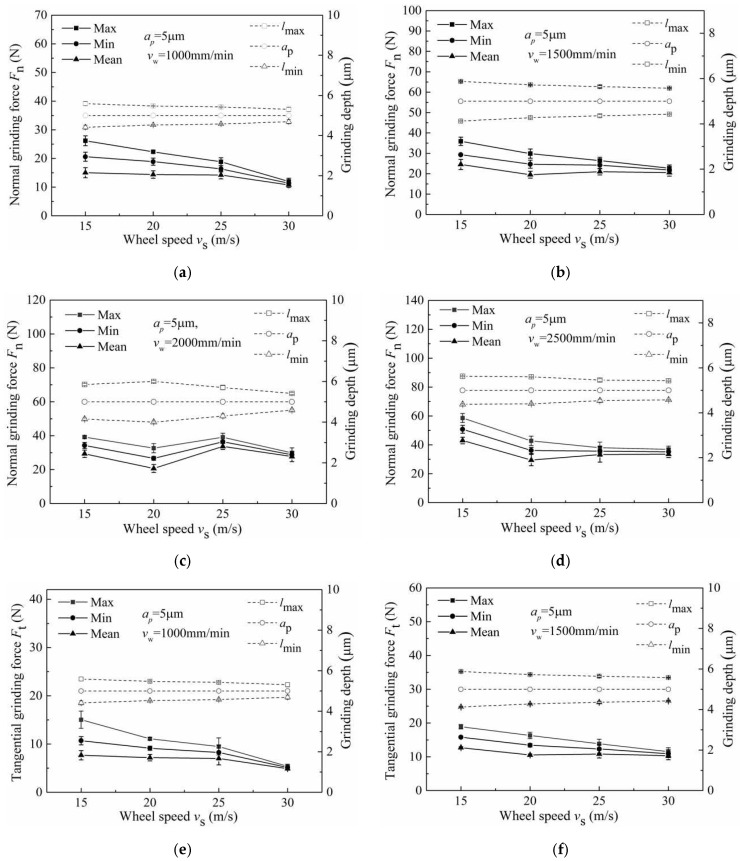

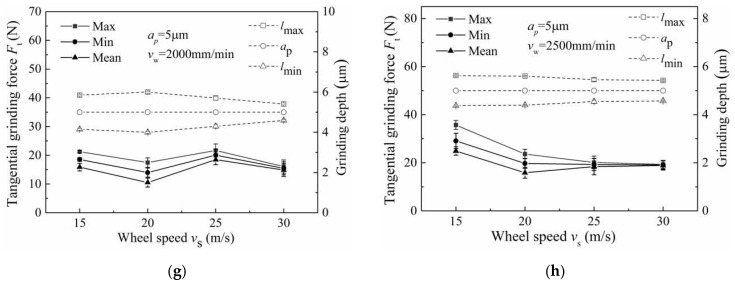
Influence of wheel speed on (**a**–**d**) normal grinding force and grinding depth and (**e**–**h**) tangential grinding force and grinding depth.

The grinding signals before and after filtering are shown in Figure 7a,b. In the following figures, the max, min and mean represent the peak value, valley value and the average value of the grinding signal, respectively. *l*_max_, *a*_p_ and *l*_min_ represent the actual maximum grinding depth, theoretical grinding depth and the actual minimum grinding depth, respectively. The investigations of grinding force were carried out under the following machining conditions: worktable feed of 1000 mm/min, 1500 mm/min, 2000 mm/min and 2500 mm/min, as shown in Figure 8. Here, the grinding depth was 5 μm, and the wheel speed ranged from 15 m/s to 30 m/s. 

It can be seen from Figure 8 that the grinding force decreased with an increase in wheel speed (Figure 8c,g shows some fluctuations); this was mainly caused by the decrease in the maximum undeformed chip thickness with the increase in wheel speed [22]. For this characteristic, the maximum and mean curves increase relative to the minimum curve. The difference between the max curves and the min curves decreased with the increase in wheel speed and was consistent with the difference between the *l*_max_ curves and the *l*_min_ curves, which also varied with the wheel speed. This was mainly caused by the decrease in wheel runout with the increase in wheel speed.

**Figure 9:**
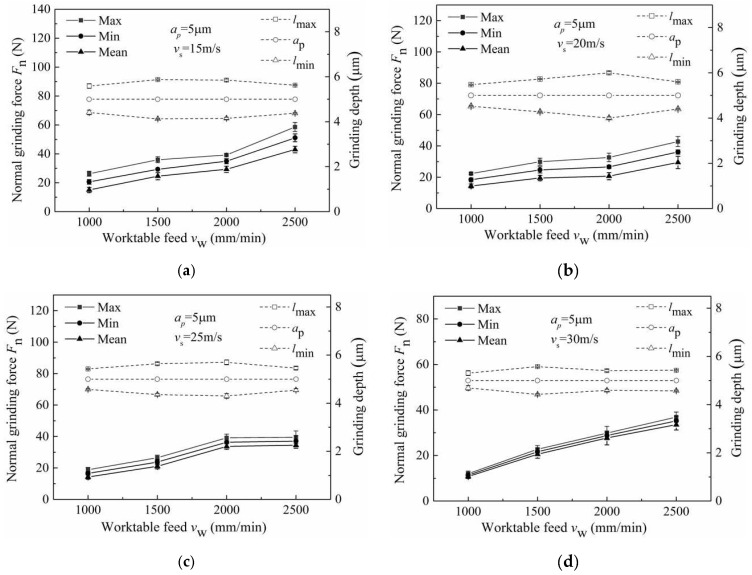

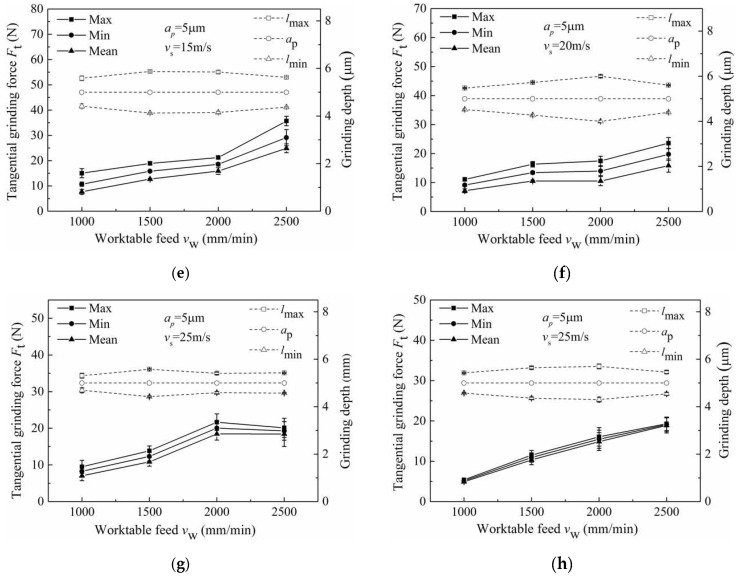
Influence of worktable feed on (**a**–**d**) normal grinding force and grinding depth and (**e**–**h**) tangential grinding force and grinding depth.

The investigation of grinding forces was carried out under the following machining conditions: wheel speed of 15 m/s, 20 m/s, 25 m/s and 30 m/s, as shown in Figure 9. Here, the depth of cut was fixed at 5μm and the worktable feed ranged from 1000 mm/min to 2500 mm/min. As can be seen from Figure 9, the grinding force increased with an increase worktable feed (Figure 9g shows some fluctuations); this was mainly caused by the maximum undeformed chip thickness, which increased with an increase in worktable feed [23]. For these characteristics, the maximum curve, average curve and minimum curve showed little difference. The worktable feed had little effect on the difference between the max curves and the min curves, consistent with the difference between the *l*_max_ curves and the *l*_min_ curves, which varied with the worktable feed. This was mainly caused by the worktable feed having no effect on wheel runout.

### 3.4. Influence of Grinding Parameters on Surface Roughness

**Figure 10:**
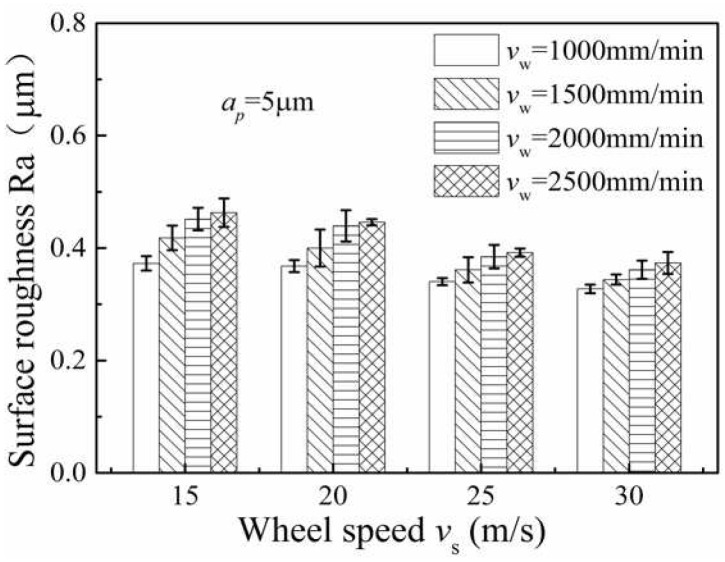
Variation of workpiece surface roughness Ra with wheel speed under different worktable feeds.

The investigation of the average value of the ground surface roughness Ra was carried out under the following machining conditions: worktable feed of 1000 mm/min, 1500 mm/min, 2000 mm/min and 2500 mm/min, as can be seen from Figure 10. Here, the depth of cut was fixed at 5 μm and the wheel speed ranged from 15 m/s to 30 m/s. The average value of ground surface roughness Ra decreased with an increase in wheel speed and increased with an increase in worktable feed; this is mainly because the maximum undeformed chip thickness of a single abrasive decreases with the increase of grinding wheel speed and the decrease of table speed. In this study, the influence of grinding parameters on the average value of roughness Ra was not obvious.

## 4. Conclusions

(1). The grinding force signal changed with the sine wave under a small cutting depth (5 μm); this was caused by grinding wheel runout.
(2). The difference of grinding force between the peak and valley was reduced by increasing the grinding wheel speed, which was mainly due to the decrease in average grinding force when the maximum undeformed cutting thickness of the single abrasive decreased. The actual height difference of the grinding surface peak and valley was realized by increasing the grinding wheel speed. In addition, the worktable feed had little effect on the difference between both.
(3). The grinding force decreased with an increase in wheel speed. The average curve and the maximum curve changed more than the minimum curve. Lower grinding force was achieved by reducing the feed speed of the worktable; however, there was no significant difference in the variation of the three curves.
(4). Lower average surface roughness was achieved by reducing the feed speed and increasing the grinding wheel speed. This was mainly affected by the maximum undeformed cutting of the single abrasive particles.

## Figures and Tables

**Table 1:** Chemical composition of Fe-Cr-Co permanent magnet alloy (%).

Element	C	Si	Mn	P	S	Cr	Co	Fe
Percent	0.01	0.97	0.0027	0.005	0.004	24.28	12.17	Bal.

**Table 2:** Mechanical properties of Fe-Cr-Co permanent magnet alloy.

Microhardness (HV)	Tensile Strength (MPa)	Elongation Rate (%)	Reduction in Area (%)
590	680	25	41

**Table 3:** Grinding conditions.

Types	Contents
Grinding wheel type and size (mm)	PA46L; 400 × 40 × 127
Workpiece size (mm^3^)	25(length) × 16(width) × 14.5(height)
Grinding mode	Down grinding
Wheel speed *v*_s_ (m/s)	15, 20, 25, 30
Worktable feed *v*_w_ (mm/min)	1000, 1500, 2000, 2500
Depth of cut *a*_p_ (μm)	5
Grinding state	Dry grinding

**Table 4:** Comparison between the number of wheel runouts and the number of peaks in force signals.

Number	*v*_s_ (m/s)	*v*_w_ (mm/min)	*n* (rev/s)	*t* (s)	*z*	*u*
1	15	1000	11.94	0.96	11.45	12
2	20	1000	15.92	0.96	15.27	16
3	25	1000	19.89	0.96	19.09	20
4	30	1000	23.87	0.96	22.91	23
5	15	1500	11.94	0.64	7.64	8
6	20	1500	15.92	0.64	10.18	11
7	25	1500	19.89	0.64	12.73	13
8	30	1500	23.87	0.64	15.28	15
9	15	2000	11.94	0.48	5.73	7
10	20	2000	15.92	0.48	7.64	8
11	25	2000	19.89	0.48	9.55	10
12	30	2000	23.87	0.48	11.46	11
13	15	2500	11.94	0.38	4.58	5
14	20	2500	15.92	0.38	6.11	6
15	25	2500	19.89	0.38	7.64	7
16	30	2500	23.87	0.38	9.16	8

**Table 5:** Relationship between grinding forces and actual grinding depth.

Number	*n* (rev/s)	Filter Frequency (Hz)	Peak Value (N)	Valley Value (N)	Average Value (N)	*l* (μm)	*l*_max_ (μm)	*l*_min_ (μm)
1	11.94	12	26.20	15.03	20.61	1.18	5.59	4.41
2	15.92	16	22.32	14.40	18.36	0.95	5.48	4.53
3	19.89	20	18.84	14.25	16.54	0.85	5.43	4.58
4	23.87	24	11.98	10.77	11.38	0.62	5.31	4.69
5	11.94	12	35.90	24.55	30.22	1.75	5.88	4.13
6	15.92	16	29.85	19.52	24.68	1.45	5.73	4.28
7	19.89	20	26.45	21.00	23.73	1.28	5.64	4.36
8	23.87	24	22.70	20.56	21.63	1.15	5.58	4.43
9	11.94	12	39.16	29.33	34.25	1.9	5.95	4.05
10	15.92	16	32.62	20.66	26.64	1.58	6.00	4.00
11	19.89	20	39.08	33.69	36.38	1.03	5.52	4.48
12	23.87	24	29.81	27.71	28.76	0.82	5.41	4.59
13	11.94	12	58.56	43.03	50.80	1.25	5.63	4.38
14	15.92	16	42.77	29.47	36.12	1.2	5.60	4.40
15	19.89	20	38.00	33.31	35.66	0.91	5.46	4.54
16	23.87	24	36.86	33.53	35.20	0.85	5.43	4.58
